# Beyond the game: athletic identity and post-retirement adaptation of former Major League Baseball players

**DOI:** 10.3389/fpsyg.2026.1810174

**Published:** 2026-07-29

**Authors:** Mark Worrell, Xiaofen D. Hamilton, Jiren Zhang, Yucen Li, Timothy Lynch

**Affiliations:** 1 Department of Exercise and Sport Studies, Warner University, Lake Wales, FL, United States; 2 Department of Curriculum and Instruction, The University of Texas at Austin, Austin, TX, United States; 3 School of Physical Education, Huaqiao University, Quanzhou, China; 4 Department of Education, Yew Chung College of Early Childhood Education, Hong Kong, Hong Kong SAR, China; 5 Yew Chung International School of Chongqing, Yew Chung Yew Wah Education Network, Chongqing, China

**Keywords:** athletic identity, athletic identity loss, role identity, sport retirement, career transition, professional athletes, Major League Baseball, AIMS-Plus

## Abstract

**Purposes:**

The transition from professional athletics to retirement can be particularly complex for Major League Baseball (MLB) players, whose athletic identity is often formed early and reinforced throughout long and demanding careers. This study examined how retired MLB players perceive their athletic and role identities following retirement and explored differences among individuals experiencing high, moderate, and low levels of athletic identity loss.

**Method:**

Data were collected from 194 retired MLB players using the third version of the Athletic Identity Measurement Scale Plus (AIMS-Plus) and a role identity rating scale. Most of the participants were White (80.9%) with the mean age of 53 years. Participants were categorized into high, moderate, and low athletic identity loss groups based on their Total AIMS (TAIMS) scores. A multivariate analysis of covariance (MANCOVA) was conducted to examine group differences with MLB service time included as a covariate.

**Results:**

The MANCOVA revealed a significant multivariate effect of athletic identity loss level on the combined AIMS-Plus subscales after controlling for MLB service time with a large effect size. MLB service time also demonstrated a small but statistically significant multivariate effect. Bonferroni-adjusted *post hoc* analyses demonstrated a clear gradient pattern: participants in the low athletic identity loss group scored significantly higher than those in the moderate and high groups across all AIMS-Plus subscales and the moderate group scored significantly higher than the high group across all AIMS-Plus subscales. In contrast, no significant differences were observed across athletic identity loss groups for most role identity variables, except the role of athletics. No significant differences were observed across athletic identity loss groups for the remaining role identity variables.

**Conclusions:**

These findings underscore the strong association between athletic identity loss and multiple dimensions of athletic identity among retired MLB players. The results highlight the importance of early identity exploration and career planning in mitigating the psychological impact of retirement. Additionally, the study emphasizes the need for intentional mentorship and transition programs within MLB to support athletes in preparing for life after baseball and promoting long-term wellbeing.

## 1 Introduction

Professional athletes are often recognized for their success and public visibility; however, retirement from sport can introduce significant psychological and emotional challenges. Athletes who are forced into involuntary retirement may experience a significant disruption to a highly salient role that has long served as a central component of their personal and athletic identity (Ronkainen et al., 2023; Schmid et al., 2024). Research has consistently shown that retired athletes may experience depression, anxiety, and loss of purpose during this transition (Voorheis et al., 2023; Mesquita Garganta da Silva et al., 2024; Runacres and Marshall, 2024). Despite these findings, public narratives continue to emphasize success while often overlooking the long-term psychological demands associated with elite sport participation (Zhang, 2024; Gaetz, 2021).

Retired Major League Baseball (MLB) players represent a unique and understudied population due to prolonged developmental pathways, early professional entry, and sustained immersion in highly structured environments. MLB players typically enter professional systems at a young age and progress through extended developmental pathways, often spending years within minor league structures before reaching the major league level. This prolonged immersion may strengthen identification with the athlete role while limiting opportunities to develop alternative identities. Consequently, retirement may represent a more complex identity transition, particularly when it occurs due to injury, deselection, or other uncontrollable factors (Souter et al., 2018; Rajaram, 2021). Despite growing research on athletic identity and retirement, retired MLB players remain an understudied population.

Measurement of athletic identity has advanced through instruments such as the Athletic Identity Measurement Scale (AIMS) and the AIMS-Plus (Cieslak, 2004; Brewer et al., 2022). The AIMS examines the salience of the athlete role, while the AIMS-Plus expands this framework by incorporating both internal dimensions, such as self-perception and emotional attachment, and external influences, including family, peers, and media (Fitriana and Xin, 2019). These tools provide a structured approach to examining how athletic identity is formed and maintained across contexts (Cabrita et al., 2014; Monteiro et al., 2021).

Examining athletic identity alongside role identity provides a more comprehensive understanding of retirement experiences. Identity theory suggests that individuals maintain multiple role-based identities that vary in salience and are reorganized as life circumstances change (Grimell, 2024). For professional baseball players, the athlete role often dominates due to early specialization and sustained immersion in sport (Gentile et al., 2025). The Role Identity Scale (Lochbaum et al., 2022) complements athletic identity measures by assessing how individuals define themselves across roles following retirement. Together, these approaches allow for a more precise examination of identity continuity, disruption, and adaptation.

With respect to the factors influencing athletic identity, age is an important consideration because athletes' identification with the athlete role often evolves across the lifespan and throughout the course of their athletic careers (Schmid et al., 2024). Previous research involving athletes from sports other than baseball has shown that athletic identity tends to decline as athletes spend more time in their sport and progress through adulthood (Houle and Kluck, 2015; Schmid et al., 2024). This decline is often attributed to the increasing responsibilities associated with other life roles, such as family, employment, and career development, which contribute to a more diversified sense of identity beyond athletics (Houle and Kluck, 2015). Athletes who maintain an exclusive or highly salient athletic identity into later adulthood may experience greater psychological distress when faced with involuntary retirement, whereas those with a more balanced self-concept often adapt more successfully to life after sport (Schmid et al., 2024). Therefore, understanding the relationship between age and athletic identity is essential for supporting athletes' long-term psychological wellbeing and facilitating successful career transitions.

Accordingly, this study is guided by the following research questions:

R1: What is the nature of retired MLB players' athletic and role identity? R2: What athletic identity and role identity differences exist between retired MLB players reporting high, medium, and low levels of athletic identity loss? R3: How does age, interact with athletic identity loss levels and role identity subscales?.

The following hypotheses were tested:

H1: Retired MLB players will report a strong and enduring athletic identity, with athletic identity remaining more salient than other role identities. H2: Athletic identity loss level (low, medium, high) will have a significant multivariate effect on athletic and role identity, such that players reporting lower athletic identity loss will demonstrate stronger identification than those reporting moderate or high athletic identity loss. H3: Age will significantly moderate the relationship between athletic identity loss level and identity outcomes.

By integrating athletic identity theory with role identity theory, this study advances understanding of how sport-specific structures shape identity transition following career termination. The findings offer practical implications for supporting athletes in managing athletic identity loss and facilitating adjustment beyond sport.

## 2 Methods

### 2.1 Research design

A total of 194 retired Major League Baseball players recruited through the Major League Baseball Players Alumni Association (MLBPAA) completed an online survey that included the third version of the Athletic Identity Measurement Scale Plus (AIMS-Plus) (Cieslak, 2004; Cabrita et al., 2014), a validated and reliable instrument for assessing athletic identity. The survey also included a role identity scale (Cieslak, 2004; Palermo et al., 2023) and demographic and contextual information, including age, race, and years of MLB service. This study was approved by the Institutional Review Board Chair—The University of Texas at Austin.

### 2.2 Participants and recruitment

Following Institutional Review Board (IRB) approval, participants were purposefully recruited from the MLBPAA, an organization of approximately 7,000 members, including former and current MLB players, umpires, coaches, and other baseball personnel. Eligibility for participation in this study required individuals to be former MLB players who had played in at least one official MLB game. A recruitment letter was mailed to 400 eligible MLBPAA members. In response, 210 individuals contacted the primary researcher expressing interest in the study. After obtaining informed consent, 194 of these participants completed the online survey. The 210 responses (i.e., more than 50% of the eligible members), and specifically the 194 completed surveys are considered both adequate and representative of the population of retired MLB players, especially considering the difficulty of accessing this unique group (Bullock, 2021). Given the specificity of this population and the challenges involved in recruiting retired professional athletes, the sample provides valuable insight into the experiences of former MLB players.

The mean age of participants was 53 ± 14.4 years. The ethnic breakdown was 80.9% Caucasian American, 8.2% African American, 7.7% Hispanic/Latino American, and 0.3% Other. Because of the small percentage of participants in various ethnic groups, all of them were regrouped as the minority group (i.e., 29.1%). On average, participants played 6.29 years in the Major Leagues and 5.49 years in the Minor Leagues or independent baseball (see Table 1).

**Table 1:** Demographics of retired major league baseball (MLB) survey participants.

	Variable	*n*	%
Age range	30-44	64	33.0
	45–59	60	30.9
	60+	70	36.1
Race	African American	16	8.2
	Caucasian	157	80.9
	Hispanic	15	7.7
	Other	6	3.1
MLB service	0–4 years	103	53.9
	5–9 years	49	25.7
	10–14 years	36	18.8
	15 + years	3	1.6
Service in minor league	0–4 years	61	31
	5–9 years	106	54.6
	10–14 years	24	12.4
	15 + years	3	1.5

### 2.3 Measures and instrument

#### 2.3.1 AIMS-plus and variables

The AIMS-Plus is grounded in a theoretical model that recognizes two core dimensions influencing identity: internal components, which shape the athlete's self-perception and emotional experiences, and external components, reflecting the impact of social influences such as family, coaches, peers, and media on the athlete's identity (Fitriana and Xin, 2019). The AIMS-Plus consists of 22 items divided into five sections, (a) three variables assess internal components: self-identity, positive affectivity, and negative affectivity, (b) two variables assess external components: social identity and exclusivity, shaped by the surrounding environment and relationships. Each item is rated using a 0–100 Likert scale (0 = totally disagree; 100 = totally agree), along with a demographic information section, which consisted of commonly measured variables such as age, and sex.

AIMS-Plus is a validated self-report instrument designed to assess the strength, importance, and exclusivity of athletic identity (Cieslak, 2004; Palermo and Rancourt, 2019; Monteiro et al., 2021). AIMS-Plus was designed to broaden the scope of the original AIMS by incorporating additional dimensions of athletic identity (Brewer at al., 2022). While the original measure primarily emphasized social identity, exclusivity, and negative affectivity, AIMS-Plus also includes constructs such as positive affectivity and self-identity, offering a more comprehensive approach to understanding athletes' self-concept (Cieslak, 2004; Cabrita et al., 2014).

Research on the scale's reliability indicates solid performance. Initial testing demonstrated adequate internal consistency across the five subscales, with Cronbach's alpha coefficients supporting its stability (Cieslak, 2004). Follow-up adaptations in international settings, including a Portuguese version replicated these findings, suggesting that the instrument can yield reliable outcomes across different cultural contexts (Cabrita et al., 2014; Clark et al., 2025). Although no published study has formally validated the AIMS-Plus exclusively in retired MLB players, the scale has been successfully used in populations closely related to the present sample. The AIMS-Plus has demonstrated its reliability and construct validity in multi-sport athlete samples, including football, basketball, swimming, and gymnastics (Cabrita et al., 2014). In addition, the scale has been examined in samples of current and former collegiate and professional athletes from team-based sports such as basketball and ice hockey, further supporting its factor structure and applicability in competitive sport contexts (Palermo et al., 2023). Beyond active athletes, the AIMS has also been used in retired elite athletes from sports including soccer, handball, and track and field, demonstrating its relevance for assessing identity changes during sport career transitions (Schmid et al., 2024). Furthermore, the exiating evidence indicates that the AIMS and its extended versions have been widely applied across diverse athletic populations, encompassing both individual and team sports as well as varying levels of competition (Lochbaum et al., 2022).

#### 2.3.2 Role identity scale and measures

Role identity was assessed using a role identity rating approach grounded in identity theory, which conceptualizes identity as composed of multiple roles that vary in importance and salience within the self-concept (Gallagher et al., 2022). Rather than treating identity as a singular construct, this approach captures the relative strength individuals assign to different life roles, allowing for examination of how identity priorities shift following major life transitions.

In the present study, participants were asked to rate the importance of several identity domains, including athletics, family, friendships, work or career, religion, and romantic relationships measured by the Role Identity Scale developed by Cieslak (2004). These ratings were used to assess the centrality of the athlete role relative to other meaningful roles in participants' lives following retirement from MLB. Higher ratings for athletics relative to other domains were interpreted as stronger role identity centrality, whereas lower ratings suggested greater disengagement from the athlete role and broader identity reconstruction. Prior research supports the use of role identity ratings as a valid method for distinguishing individuals who experience greater difficulty disengaging from a dominant prior role during career transitions (Zvosec et al., 2023; Haslam et al., 2024). Within the context of professional sport retirement, this approach allows for a nuanced examination of athletic identity loss by capturing not only attachment to the athlete role, but also the extent to which alternative roles have been adopted or strengthened. When used in conjunction with the AIMS Plus, the Role Identity Scale provides a complementary perspective by situating athletic identity within the broader constellation of life roles, thereby enhancing the study's ability to examine identity disruption, continuity, and adaptation among retired MLB players.

#### 2.3.3 Response scale and measurement approach

A 0–100 response scale was used to assess both athletic identity loss and role identity. Although less common than traditional Likert formats, this scale was selected for several methodological reasons. A 0–100 (i.e., 101-point in total) scale more closely approximates a continuous measure than conventional 5- or 7-point Likert scales, thereby enhancing sensitivity to detect fine-grained differences in respondents' attitudes and self-perceptions (Couper et al., 2006; Lewis and Erdinç, 2017). In addition, scales with a larger number of Preston response categories have been shown to improve measurement precision, reliability, and discriminating power while reducing information loss (Preston and Colman, 2000; Lozano et al., 2008). Such formats may also produce distributions that better approximate normality, which is advantageous for parametric statistical analyses. The broader response range allows for more precise distinctions in perceived identity strength, which is particularly important when examining nuanced constructs such as athletic identity loss, and helps reduce ceiling and floor effects in professional athlete populations (Lochbaum et al., 2022). Furthermore, the 0–100 format has been consistently used in the development and validation of the AIMS and its extended versions, demonstrating strong psychometric properties across diverse athlete samples (Cieslak, 2004; Cabrita et al., 2014; Lochbaum et al., 2022). Accordingly, this format was retained to maximize measurement precision and maintain consistency with prior research (Brewer et al., 2022).

The Role Identity Scale was included to assess the extent to which individuals continue to identify with various non-sport roles following retirement. This measure complements athletic identity assessments by capturing broader dimensions of self-concept across personal, social, and professional domains (Silva et al., 2020). Using both measures together allows for a robust evaluation of identity restructuring, providing insight into not only the presence of athletic identity loss, but also the extent to which alternative role identities are maintained or developed (Bejar et al., 2026). This combined approach strengthens the conceptual alignment between athletic identity loss and role identity frameworks, offering a nuanced understanding of identity transition among retired MLB athletes.

### 2.4 Data analysis

Following the completion of the data screening, Harman's single factor test (Podsakoff et al., 2024) was conducted to assess the potential presence of common method bias across all self-report measures included in the study. Both the AIMS Plus items and the role identity rating items were entered simultaneously into an unrotated exploratory factor analysis to evaluate whether a single latent factor accounted for the majority of shared variance among the measures. Several criteria were examined, including the Kaiser–Meyer–Olkin measure of sampling adequacy, Bartlett's test of sphericity, the eigenvalue associated with the first factor, and the percentage of total variance explained by that factor (Shrestha, 2021). Evidence of common method bias would be indicated if one factor emerged as dominant or accounted for a disproportionate amount of variance (i.e., > 50%).

In addition to addressing common method bias, descriptive statistics were computed separately for the AIMS Plus subscales and the Role Identity Scale items to examine central tendencies and variability across identity domains. Role identity was operationalized as the mean of six role importance ratings reflecting the perceived importance of athletics, family, friendships, work or career, religion, and romantic relationships. This approach allowed for direct comparison of the relative centrality of the athlete role within the broader constellation of life roles following retirement. By including role identity ratings alongside athletic identity measures, the analysis captured both the strength of identification with the athlete role and the extent to which alternative roles had been adopted or prioritized (Lochbaum et al., 2022).

Composite scores were calculated for the survey variables. The total Athletic Identity Measurement Scale score (TAIMS) was computed as the mean of five subscales: self-identity, positive affect, negative affect, social identity, and exclusivity. Each subscale score was calculated as the mean of its constituent items. Participants were classified into three athletic identity groups based on tertile splits (33^rd^ and 66^th^ percentiles) of their total TAIMS scores: low athletic identity loss (≥ 67.39, *n* = 64), medium athletic identity loss (42.55–67.38, *n* = 64), and high athletic identity loss (≤42.54, *n* = 66).

To examine identity level group and age differences in AIMS-Plus subscales, and role identity variables, multivariate analyses of covariance (MANCOVAs) were conducted. Prior to conducting the MANCOVA, assumptions of multivariate normality and homogeneity of covariance matrices were evaluated. For variables in athletic identity scale, Box's M test indicated a significant violation of the assumption of equal covariance matrices across groups, (Box's *M* = 304.15, *F*_(30, 115412.48)_ = 9.76, *p* < 0.001). Therefore, Pillai's Trace was used to evaluate multivariate effects (Meyers et al., 2017). TAIMS was entered as a fixed factor, and MLB service time was included as a covariate in all models. When significant multivariate effects were detected, follow-up univariate analyses of covariance (ANCOVAs) were performed to identify differences across individual outcome variables.

Similarly, assumptions for the MANCOVA on the six role identity items, controlling for service time, were assessed. Box's M test indicated violation of covariance equality for Role Identity variables (Box's *M* = 86.42, *F*_(42, 108134.21)_ = 1.97, *p* < 0.001), therefore Pillai's Trace was used. Effect sizes were reported as partial eta squared (η^2^) with values of 0.01,0.06, and 0.14, indicating small, medium, and large effects, respectively (Meyers R. et al., 2017). The statistical significance was set at *p* < 0.05. All data analysis was performed using SPSS 27.0.

## 3 Results

To assess potential common method bias, Harman's single-factor test was conducted using an unrotated principal component analysis on all measurement items from the AIMS-Plus subscales and the Role Identity scale. The Kaiser–Meyer–Olkin measure of sampling adequacy was 0.930, and Bartlett's test of sphericity was significant [χ^2^_(378)_ = 4,671.047, *p* < 0.001], indicating that the data were suitable for factor analysis. The unrotated solution revealed multiple factors, with the first factor accounting for 46.68% of the total variance. The eigenvalue of the first factor is 13.07. These findings indicate that common method bias is unlikely to pose a significant concern. However, given the limitations of Harman's single-factor test, the results should be interpreted with caution.

### 3.1 Differences of AIMS-plus subscales among athletic identity level groups and age groups

After controlling for service time, the multivariate effect of TAIMS identity level on the combined AIMS-Plus subscales was significant (Pillai's Trace = 1.014, *F*_(10,374)_ = 38.47, *p* < 0.001, η^2^ = 0.507). MLB service time also demonstrated a small but statistically significant multivariate effect (Pillai's Trace =0.060, *F*_(5,186)_ = 2.37, *p* < 0.05, η^2^ =0.060). Follow-up univariate ANCOVAs revealed significant main effects of TAIMS identity level on all AIMS-Plus subscales, including self-identity, positive affect, negative affect, social identity, and exclusivity (all *p* < 0.001). Effect sizes were large across all subscales (partial η^2^ ranged from 0.593 to 0.730). Bonferroni *post-hoc* tests indicated that for every subscale, the low athletic identity loss group scored significantly higher than both the moderate- and high athletic identity loss groups, and the moderate athletic identity loss group scored significantly higher than the high athletic identity loss group (see Table 2). In terms of age differences in AIMS-Plus subscales, age group showed a significant multivariate effect after controlling for MLB service time (Pillai's Trace = 0.097, *F*_(10,374)_ = 1.91, *p* = 0.043, η^2^ = 0.048). Follow-up ANC OVAs showed significant age-group effects for social identity (*F*_(2,190)_ = 3.56, *p* = 0.030, η^2^ = 0.036), and exclusivity (*F*_(2,190)_ = 3.67, *p* = 0.027, η^2^ = 0.037). Pairwise comparisons indicated that the middle-aged group scored significantly higher than the older group on exclusivity, whereas no significant pairwise differences emerged for social identity (see Table 3).

**Table 2:** Differences in AIMS-plus subscales among athletic identity loss level groups.

Variable	^a^High athletic identity loss	^b^Medium athletic identity loss	^c^Low athletic identity loss	*F*_(2,190)_	*η^2^*
	(*n* = 64)	(*n* = 64)	(*n* = 66)		
	*M* (SD)	*M* (SD)	*M* (SD)		
Self-identity	24.10 (17.96) ^ab**ac**^	62.77 (22.72) ^bc**^	92.27 (8.49)	257.3^**^	0.73
Positive affectivity	45.00 (24.30) ^ab**^ ^ac**^	82.70 (19.85) ^bc**^	97.31 (6.14)	138.39^**^	0.593
Negative affectivity	18.71 (16.71) ^ab**^ ^ac**^	49.92 (26.27) ^bc**^	82.20 (1.13)	183.72^**^	0.659
Social identity	2.28 (12.31) ^ab**^ ^ac**^	41.66 (16.41) ^bc**^	73.47 (11.93)	249.25^**^	0.724
Exclusivity	13.25 (12.80) ^ab**^ ^ac**^	32.00 (16.19) ^bc**^	63.15 (19.97)	153.32^**^	0.617

^**^ indicated significant at *p* < 0.001. Superscript letters indicate significant pairwise differences among groups (a = high identity loss, b = medium identity loss, c = low identity loss).

**Table 3:** Differences in AIMS-plus subscales among age groups.

Variable	^a^Young age group	^b^Middle-aged group	^c^Older age group	*F*_(2,190)_	*η^2^*
	(*n* = 64)	(*n* = 60)	(*n* = 70)		
	*M* (SD)	*M* (SD)	*M* (SD)		
Self-identity	66.33 (31.07)	61.00 (36.30)	53.50 (30.71)	2.46	
Positive affectivity	78.16 (25.86)	74.83 (33.11)	72.89 (27.18)	0.54	
Negative affectivity	57.46 (32.63)	49.67 (34.50)	45.14 (28.56)	2.57	
Social identity	49.58 (25.66)	48.63 (27.65)	38.89 (23.39)	3.56^*^	0.036
Exclusivity	38.38 (27.25)	41.93 (27.46)^bc*^	29.89 (23.83)	3.67^*^	0.037

^*^ indicated significant at *p* < 0.05. Superscript letters indicate significant pairwise differences among groups [a = young age group (≤44 years), b = middle-aged group (45–59 years), and c = older age group (≥ 60 years)].

### 3.2 Differences of role identity subscales among athletic identity loss level groups and age groups

TAIMS athletic identity loss Level had a significant multivariate main effect on role identity subscales (Pillai's Trace = 0.502, *F*_(12,370)_ = 10.40, *p* < 0.001, η^2^ =0.251). The effect of the covariate MLB service time was not significant (Pillai's Trace = 0.040, *F*_(6,184)_ = 1.30, *p* = 0.260). Univariate results indicated a significant main effect of TAIMS was found only for identity of Athletics (*F*_(2,190)_ = 74.43, *p* < 0.001, η^2^ = 0.439). *Post-hoc* tests confirmed that the low athletic identity loss group scored significantly higher than the moderate- and high athletic identity loss groups, and the moderate-athletic identity loss group scored higher than the high athletic-identity group on identity of Athletics (all *p* < 0.001). No other role identity variables showed significant differences across the athletic identity level groups (see Table 4). For role identity domains, age group showed a significant multivariate effect after controlling for MLB service time (Pillai's Trace =0.222, *F*_(12,370)_ = 3.85, *p* < 0.001, η^2^ = 0.111), whereas MLB service time was not significant, Pillai's Trace =0.059, *F*_(6,184)_ = 1.91, *p* = 0.081, η^2^ = 0.059. Follow-up ANCOVAs showed significant age-group effects for family(F_(2,190)_ = 4.57, *p* =0.012, η^2^ =0.046); athletics (*F*_(2,190)_ = 9.32, *p* < 0.001, η^2^ = 0.090); and romance (*F*_(2,190)_ = 16.94, *p* < 0.001, η^2^ =0.152). Pairwise comparisons indicated that the young group scored higher than the older group on family, and both the young and middle-aged groups scored higher than the older group on athletics and romance (see Table 5).

**Table 4:** Differences of role identity subscales between athletic identity loss level groups.

Variable	^a^High athletic identity loss	^b^Medium athletic identity loss	^c^Low athletic identity loss	*F*_(2,190)_	*η^2^*
	(*n* = 64)	(*n* = 64)	(*n* = 66)		
	*M* (SD)	*M* (SD)	*M* (SD)		
Family	95.69 (11.05)	98.20 (6.86)	97.09 (7.54)	1.37	
Friendships	84.50 (18.42)	87.39 (14.22)	86.97 (14.06)	0.73	
Athletics	43.41 (20.66) ^ab**ac**^	64.06 (20.91) ^bc**^	84.53 (15.82)	74.43^**^	0.439
Academics	49.48 (24.70)	56.88 (24.66)	49.77 (26.10)	2.01	
Religion	87.02 (20.51)	85.86 (22.46)	86.85 (24.30)	0.07	
Romance	70.20 (28.47)	78.73 (18.31)	78.61 (22.03)	2.81	

^**^ indicated significant at *p* < 0.001. Superscript letters indicate significant pairwise differences among groups (a = high athletic identity loss, b = medium athletic identity loss, c = low athletic identity loss).

**Table 5:** Differences of role identity subscales among age groups.

Variable	^a^Young age group	^b^Middle-aged group	^c^Older age group	*F*_(2,190)_	*η^2^*
	(*n* = 64)	(*n* = 60)	(*n* = 70)		
	*M* (SD)	*M* (SD)	*M* (SD)		
Family	98.64 (3.90)^ac*^	98.03 (5.33)	94.57 (12.79)	4.57^*^	0.046
Friendships	87.73 (14.25)	86.03 (14.46)	85.00 (17.82)	0.74	
Athletics	71.25 (23.16)^ac**^	67.95 (25.85)^bc*^	54.47 (24.77)	9.32^**^	0.09
Academics	56.33 (25.65)	53.39 (27.27)	47.03 (22.72)	2.61	
Religion	82.80 (27.28)	88.73 (19.33)	88.03 (19.64)	1.004	
Romance	82.31 (18.76)^ac**^	82.27 (18.37)^bc**^	64.26 (26.90)	16.94^**^	0.152

^*^ indicated significant at *p* < 0.05, ^**^ indicated significant at *p* < 0.001. Superscript letters indicate significant pairwise differences among groups [a = young age group (≤44 years), b = middle-aged group (45–59 years), and c = older age group (≥ 60 years)].

## 4 Discussion

Competing at the highest level of baseball is both a grueling and deeply rewarding experience. For many, stepping onto a Major League field for the first time is the fulfillment of a lifelong dream born in childhood (Kaha'i, 2022; Rader, 2025). This passion for the game of baseball drives players to pursue excellence whether through personal milestones, team victories, or career achievements (Sigmundsson et al., 2022). Over time, professional baseball becomes more than an occupation; it provides structure, meaning, social belonging, and a primary framework through which professional baseball players understand themselves and their place in the world (Gentile et al., 2025). As a result, the athlete role is continuously reinforced through organizational routines, public recognition, performance evaluation, and peer culture, making it highly prominent and deeply internalized. While this immersion fuels elite performance, it also creates conditions in which identity becomes closely tethered to athletic participation, rendering the transition away from professional baseball particularly complex when that role is disrupted through retirement (Senecal and Williams, 2024).

This study aimed to explore how former MLB players perceive their athletic identity and how those perceptions have evolved since leaving professional baseball. By addressing the research questions, the goal was to provide meaningful insights into the shifting nature of athletic identity among retired players. The findings are intended to illuminate the experiences of former MLB athletes, offering a deeper understanding of identity transformation post-retirement. The results provide strong evidence that athletic identity loss remains a central and enduring issue for retired MLB athletes and that differences in athletic identity loss are meaningfully associated with athletes' self-perceptions, emotional experiences, and social identification following retirement. These findings advance the development of targeted strategies to facilitate smoother transitions among retired professional baseball players into post-career life.

### 4.1 Athletic identity and identity loss after retirement

Previous research suggests that expressing emotions and reflections related to retirement from sport can play a valuable role in the overall adjustment process (Schmid et al., 2024). Consistent with expectations, TAIMS athletic identity loss level demonstrated a significant multivariate main effect on role identity subscales, indicating that athletic identity loss is meaningfully associated with how retired MLB players continue to define themselves through particular roles (Schmid et al., 2024). However, follow-up univariate analyses revealed that this effect was driven exclusively by differences in the *identity of athletics* subscale. Consistent with previous research related to this topic players reporting lower levels of athletic identity loss exhibited significantly stronger identification with athletics, when compared to those reporting moderate and high athletic identity loss (Lochbaum et al., 2022; Park et al., 2023). This graded pattern suggests that athletic identity loss directly corresponds to the extent to which former players continue to view athletics as a central component of their self-concept.

These findings reinforce athletic identity theory, which posits that prolonged immersion in elite sport can lead to a highly salient and internalized athlete role that persists beyond active participation (Chen and Huang, 2025). For retired MLB players, athletic identity may remain psychologically meaningful even after career termination, particularly among those who experience minimal athletic identity loss. Conversely, players reporting higher athletic identity loss appear to disengage more fully from the athletic role, reflecting a greater degree of athletic identity restructuring following retirement. Prior research has similarly shown that athletes who experience stronger and more exclusive identification with the athlete role during their careers often report greater difficulty disengaging from that role once participation ends, particularly when retirement is unplanned or perceived as incomplete (Rajaram, 2021). Studies of elite and professional athletes indicate that sustained reinforcement of athletic identity through performance evaluation, public recognition, and organizational culture can delay identity reorientation after retirement, even when athletes are no longer actively competing (Schmid et al., 2024). Within professional baseball, long developmental pathways, extended seasons, and frequent movement across competitive levels may further entrench the athlete role, strengthening its centrality within the identity hierarchy and complicating post sport adjustment (Lewis, 2024). As a result, variations in athletic identity loss among retired MLB players may reflect differences in how effectively individuals renegotiate the meaning of athletics within their broader sense of self, rather than simple detachment from the sport.

### 4.2 Differentiation across AIMS-plus subscales

The univariate analyses further revealed that athletic identity loss was associated with large effects across all AIMS-Plus subscales, underscoring the multidimensional nature of athletic identity disruption, which has been severely neglected (Miller, 2009). Players with low athletic identity loss reported stronger self-identification as athletes, greater positive affect associated with sport participation, and stronger social identification with the athletic community. These individuals may have retained symbolic or emotional ties to baseball that allowed for continuity of identity despite career termination. In contrast, players with high athletic identity loss reported lower scores across all domains, suggesting a broader erosion of identity that extends beyond performance-related self-concept to include emotional attachment and social belonging. These patterns are consistent with prior research demonstrating that stronger and more exclusive athletic identity is associated with greater emotional attachment to sport, heightened affective responses, and sustained social identification after retirement, whereas higher levels of athletic identity loss are linked to diminished emotional connection, reduced social belonging, and more pronounced identity reorganization following career termination (Lavallee et al., 1997; Voorheis et al., 2023; Schmid et al., 2024).

Notably, the graded pattern observed across low, moderate, and high athletic identity loss groups supports the notion that athletic identity loss exists along a continuum rather than as a binary outcome (Mitchell, 2025). This finding reinforces role identity theory, which emphasizes that identities vary in salience and can be gradually reorganized rather than abruptly lost (Anglin et al., 2022). Athletes experiencing moderate athletic identity loss may be in the process of renegotiating their self-concept, whereas those with high athletic identity loss may struggle to integrate alternative roles that provide meaning and coherence. In this context, athletic identity loss should be understood not as a uniform endpoint but as part of an ongoing adjustment process in which athletes recalibrate the relative importance of the athlete role within a broader identity structure. For some former MLB players, this process may involve maintaining selective elements of athletic identity while simultaneously expanding engagement in non-sport roles, whereas for others it may require more substantial disengagement from athletics to achieve psychological balance. Recognizing athletic identity loss as a dynamic and individualized process offers a more nuanced framework for examining post sport adjustment and underscores the importance of longitudinal approaches that capture how identity evolves across different stages of retirement (Haslam et al., 2024).

### 4.3 Specificity of identity disruption

Importantly, no significant differences emerged across athletic identity loss levels for other role identity subscales. This pattern suggests that athletic identity loss does not necessarily generalize to broader role identity disruption, which is consistent with what have been reported in the literature (Stavitz et al., 2025). Rather than undermining multiple aspects of the self, athletic identity loss appears to be role-specific, primarily affecting how strongly individuals continue to identify with athletics. From a role identity perspective, this finding supports the notion that individuals hold multiple, relatively independent identities that can be reorganized without wholesale erosion of the self-concept (Collict et al., 2025).

This specificity may indicate that many retired MLB players are able to disengage from the athlete role while maintaining stability in other domains of identity. Such role differentiation may serve as a protective factor during career transitions, allowing individuals to preserve psychological coherence despite the loss of a highly salient professional role (Turner, 1975). These results align with findings reported in the literature suggesting that successful adaptation depends not solely on disengaging from sport, but on the availability and strength of alternative roles that provide meaning and continuity (Thomas et al., 2024).

Taken together, these findings suggest that the psychological impact of athletic identity loss among retired MLB players may be more contained than is often assumed (Roberts et al., 2023). Rather than triggering diffuse instability across life domains, identity disruption appears concentrated within the athletic role itself, while other salient roles remain intact (Chun et al., 2023). This pattern highlights the adaptive capacity of many former MLB players to compartmentalize identity change and selectively reorganize their identity hierarchy following retirement. In this sense, the presence of stable non-athletic roles may buffer against more pervasive identity distress, allowing former MLB players to navigate retirement with a greater sense of continuity and self-coherence. Understanding how and when such role differentiation develops may be critical for identifying factors that promote resilience during the transition away from professional sport.

These findings can also be understood within the broader literature on athletic identity and career transition, which emphasizes the importance of identity flexibility and role diversification in facilitating successful adjustment following sport retirement. Prior research has shown that athletes who develop multiple role identities during their careers tend to experience less psychological distress during transition, as they are not solely reliant on the athlete role for self-definition (Zvosec et al., 2023). The present findings extend this perspective by highlighting how identity experiences may differ within a professional baseball context, where career structure and prolonged immersion may intensify identity centrality.

From a practical standpoint, these results have direct implications for professional baseball organizations and athlete development systems. MLB teams and affiliated programs may benefit from implementing structured transition initiatives that support identity development beyond sport. This may include career planning resources, mentorship opportunities, and psychological support services aimed at helping athletes expand their sense of self while still active in their careers. Encouraging identity diversification prior to retirement may reduce the impact of athletic identity loss and promote increased positive long-term adjustment outcomes (Ybarra, 2024).

### 4.4 Role of MLB service time

MLB service time demonstrated a small but statistically significant multivariate effect on athletic identity, suggesting that length of professional experience may modestly influence post-retirement identity perceptions. While this effect was considerably smaller than that of athletic identity loss, it may reflect the cumulative impact of prolonged exposure to professional sport environments. Longer service time may intensify identification with the athlete role through sustained reinforcement, while shorter service time may limit opportunities for identity consolidation. However, the relatively small effect size indicates that service time alone does not account for the substantial variation in post-retirement identity experiences, highlighting the importance of psychological processes beyond career duration (Zhan et al., 2023).

It is surprising that MLB service time did not demonstrate a significant multivariate effect on role identity subscales. This finding suggests that MLB service time, as an objective indicator of career duration, was not significantly associated with post-retirement role identity domains in the present model. While longer MLB service time may contribute to stronger athletic identity during an athlete's career, these results indicate that athletic identity loss following retirement is influenced more by psychological processes than by objective career length. This distinction underscores the importance of examining subjective identity experiences rather than relying solely on career characteristics when assessing post-sport adjustment (Wendling and Sagas, 2025).

### 4.5 Age, athletic identity and role identity

The significant differences in athletic identity and role identity observed in the present study provide additional evidence that age is associated with selected identity dimensions among retired MLB players after controlling for MLB service time. Significant differences were found only for the social identity and exclusivity subscales of the AIMS-Plus and the family, athletics, and romance domains of the role identity scale. This pattern suggests that age-related differences were not uniform across all identity domains, but were concentrated in dimensions related to social recognition, identity exclusivity, and personally meaningful life roles. Notably, these differences were primarily observed between the youngest and oldest age groups, suggesting that age may influence specific dimensions of identity rather than overall athletic identity. Older retired players tended to report lower scores in selected athletic and role identity domains, which may indicate a gradual weakening of the athlete role and a broader reorganization of identity after retirement. One possible explanation is that, as athletes mature, they assume additional social roles and responsibilities, such as family relationships and career development, which may alter the relative importance of the athlete role (Schmid et al., 2024). In this sense, lower athletic identity among older participants may reflect not only identity loss, but also adaptive identity diversification across later adulthood. However, because previous research has not specifically examined age-group differences in these identity dimensions, the underlying reasons for these findings remain unclear. Consequently, further research is needed to investigate how athletic and role identities evolve across different stages of the lifespan and to identify the psychosocial factors that contribute to these age-related differences.

## 5 Limitations

Despite its contributions, this study is not without limitations. Like most athletic identity studies, which usually have examined small samples (Schmid et al., 2024), due to the small total number of elite athletes and difficulty of recruiting them, the sample size is relatively small for a survey study. The cross-sectional design limits causal inferences regarding the directionality of relationships between athletic identity loss and athletic identity. Longitudinal research is needed to track identity changes over time and to examine how identity reconstruction unfolds across different stages of retirement. Additionally, reliance on self-report measures may introduce response biases, and future studies could benefit from qualitative approaches that capture the lived experiences of retired athletes in greater depth.

## 6 Conclusions

This study offers valuable insight into the experiences of former MLB players and serves as a guide for current and future athletes. By preparing for life after baseball mentally, emotionally, and practically players can carry their love of the game forward while building meaningful lives beyond the baseball field.

This study demonstrates that athletic identity loss among retired MLB players is strongly associated with continued identification with athletics but does not appear to broadly disrupt other role identities. This study offers valuable insight into the experiences of former MLB players and serves as a guide for current and future athletes. These findings underscore the resilience of the broader self-concept. By clarifying how identity loss manifests within and beyond sport, this research contributes to a more nuanced understanding of athlete retirement and offers valuable implications for theory, research, and applied practice.

## 7 Implications and future directions

From a theoretical perspective, these findings extend existing literature by demonstrating the continued prominence of athletic identity long after retirement among professional baseball players. By integrating athletic identity and role identity frameworks, this study highlights how deeply embedded roles can persist even after formal participation ends, and how difficulties disengaging from these roles may impede psychological adjustment. Practically, the findings underscore the need for proactive identity-focused interventions within MLB. Programs designed to support career planning, identity exploration, and role diversification prior to retirement may mitigate athletic identity loss and promote smoother transitions. Continued involvement in baseball related roles, such as coaching or mentoring, may also help preserve aspects of the athletic self while facilitating identity integration. Sport psychologists, player associations, and organizational leaders should prioritize strategies that address identity loss alongside emotional wellbeing to support athletes across the lifespan.

Future research should explore the relationship between athletic identity loss and variables such as years played, age at retirement, and injury history. Segmenting AIMS-Plus data by cause of retirement could offer more targeted insights and support strategies. Further studies might also examine how MLB players maintain healthy relationships despite demanding schedules, or how young players can take an early approach to preparing for their future career.

Additionally, exploring mentorship programs between former and current players could serve as a a powerful potential resource. Retired players may offer guidance on both career development and life transitions, while also finding purpose in supporting the next generation. Such programs could reduce athletic identity loss, maintain players' connection to the sport, and promote psychological wellbeing for both mentors and mentees.

## Data Availability

The datasets presented in this article are not publicly available due to concerns regarding participant confidentiality and the potentially identifiable nature of the retired professional athlete sample. Requests to access the datasets should be directed to Mark Worrell, mworr277@gmail.com.
